# A DTC Niche Plexus Surrounds the Germline Stem Cell Pool in *Caenorhabditis elegans*

**DOI:** 10.1371/journal.pone.0088372

**Published:** 2014-02-19

**Authors:** Dana T. Byrd, Karla Knobel, Katharyn Affeldt, Sarah L. Crittenden, Judith Kimble

**Affiliations:** 1 Howard Hughes Medical Institute, University of Wisconsin-Madison, Madison, Wisconsin, United States of America; 2 Department of Biochemistry, University of Wisconsin-Madison, Madison, Wisconsin, United States of America; 3 Laboratory of Molecular Biology, University of Wisconsin-Madison, Madison, Wisconsin, United States of America; 4 Department of Medical Genetics, University of Wisconsin-Madison, Madison, Wisconsin, United States of America; Institute of Biosciences and Technology, Texas A&M Health Sciences Center, United States of America

## Abstract

The mesenchymal distal tip cell (DTC) provides the niche for *Caenorhabditis elegans* germline stem cells (GSCs). The DTC has a complex cellular architecture: its cell body caps the distal gonadal end and contacts germ cells extensively, but it also includes multiple cellular processes that extend along the germline tube and intercalate between germ cells. Here we use the *lag-2* DTC promoter to drive expression of myristoylated GFP, which highlights DTC membranes and permits a more detailed view of DTC architecture. We find that short processes intercalating between germ cells contact more germ cells than seen previously. We define this region of extensive niche contact with germ cells as the DTC plexus. The extent of the DTC plexus corresponds well with the previously determined extent of the GSC pool. Moreover, expression of a differentiation marker increases as germ cells move out of the plexus. Maintenance of this DTC plexus depends on the presence of undifferentiated germ cells, suggesting that germ cell state can influence niche architecture. The roles of this DTC architecture remain an open question. One idea is that the DTC plexus delivers Notch signaling to the cluster of germ cells comprising the GSC pool; another idea is that the plexus anchors GSCs at the distal end.

## Introduction

Stem cell maintenance relies on signals from the immediate microenvironment, or niche. Most stem cell niches reside directly adjacent to stem cells [1], [2] and several have extensive contact with stem cells [3]–[5]. The *Caenorhabditis elegans* gonad provides a simple and genetically tractable model for a stem cell niche. In this case, a single mesenchymal cell, the distal tip cell (DTC), is necessary and sufficient to maintain adjacent germline stem cells (GSCs) [1], [6]–[9].

**Figure 1:**
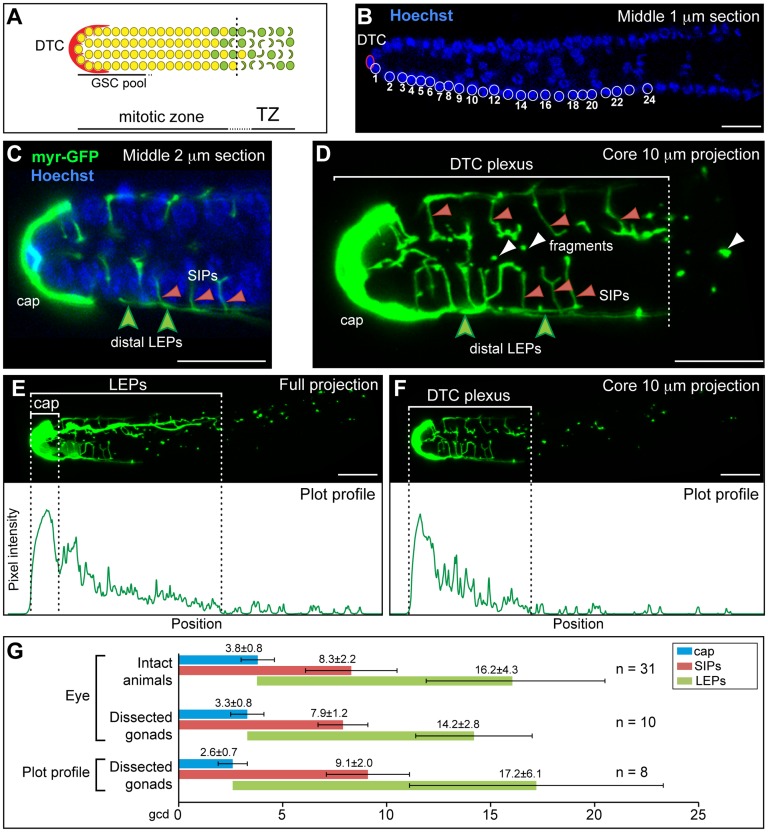
DTC architecture and the plexus region. (A) The distal gonad includes the DTC niche (red) and the mitotic zone. Yellow circles represent germ cells in the mitotic cell cycle; green circles are germ cells in meiotic S-phase; green crescents are germ cells in early meiotic prophase when chromosomes have begun to pair. Germ cells are connected by a cytoplasmic core. The mitotic zone consists of a distal stem cell pool (GSC pool) adjacent to the niche. As germ cells leave the GSC pool and progress proximally through the mitotic zone they mature. At the proximal edge of the mitotic zone, germ cells progress into early meiotic prophase as they move into the transition zone (TZ). (B) Germ cell position is scored in Hoechst-stained extruded gonads as number of germ cell diameters (gcd) from the distal end. The DTC nucleus (DTC) is oval (red oval); germ cell nuclei are round (white circles). (C) P*_lag-2_::myr-GFP* (myr-GFP, green) allows visualization of DTC membranes. This view through the central region of the gonad shows the DTC capping the distal end (cap) and short intercalating processes (SIPs; red arrowheads) on either side of germ cell nuclei (Hoechst, blue). Figure 1C modified from Byrd and Kimble [9]. (D) This “core” projection of central sections (10 µm) of a confocal z-series shows the DTC plexus, which includes the cap, short intercalating processes (SIPs; red arrowheads), long external processes (distal LEPs; green arrowheads) and internal fragments (white arrowheads). (E) The DTC cap and LEPs are best visualized in a full projection of a z-series. The cap and LEPs are detected quantitatively using Plot profile. The graph below shows pixel intensity (y-axis) along the distal-proximal axis (x-axis). Dotted lines mark the distal end of the gonad and the proximal boundary of the cap (end of initial peak of GFP fluorescence) or LEPs (first position where pixel intensity reaches background). (F) The DTC plexus is best visualized in a “core” projection of the central ten 1 µm slices of a z-series. The plexus is detected quantitatively using Plot profile. The graph below shows pixel intensity (y-axis) along the distal-proximal axis (x-axis). Dotted lines mark the distal end of the gonad and the proximal boundary (first position where the pixel intensity reaches background) of the plexus. (B–F) Scale bars = 10 µm. (G) DTC features measured by three different methods give similar results. First, live animals were scored by eye using wide-field fluorescent microscopy. Second, dissected gonads were scored by eye using wide-field fluorescent microscopy. Third, dissected gonads were scored quantitatively using confocal microscopy and Plot profile. Bar graphs show average lengths of DTC features. Error bars indicate standard deviation.

The adult *C. elegans* germline includes a pool of ∼50–75 GSCs in an undifferentiated and proliferative state [8], [10]; the DTC and GLP-1/Notch signaling are required to maintain this state [7], [8]. This GSC pool is part of a larger group of ∼225 mitotically dividing germ cells that extend proximally from the DTC and constitute the “mitotic zone” [11]. Germ cells are interconnected by a cytoplasmic core; however, germ cells in the mitotic zone are heterogeneous with respect to cell cycle, expression of key regulators and differentiation potential [12]–[14]. The GSC pool resides in the distal part of the mitotic zone (near the DTC), and is maintained in an undifferentiated state [8] (Figure 1A). By contrast, germ cells in the proximal mitotic zone (away from the DTC) have been triggered to differentiate: they exist in a gradient of maturation with least mature bordering the GSC pool and most mature bordering overt entry into the meiotic cell cycle. As germ cells divide and move proximally, they ultimately leave the mitotic zone and enter the “transition zone”, where they enter early stages of meiotic prophase (Figure 1A). In addition to its role in GSC maintenance via Notch signaling, the DTC transmits nutritional signals to the germline [15] and regulates oocyte size [16].

Previous work identified the main features of DTC architecture using both transmission electron microscopy [17], [18] and fluorescence light microscopy [17], [19]–[21]. The DTC cell body caps the distal germline and sends processes proximally; short intercalating processes (SIPs) embrace germ cells adjacent to the DTC just under the cap [18], [19]; long external processes extend proximally down the gonad with varying lengths, often beyond the mitotic zone [17], [19], [20], and detached DTC fragments exist inside the germline tissue [17], [22]. A rough correlation was suggested between the extent of DTC long processes and the boundary between mitotic and transition zones in young adults [20], but more in-depth studies showed that DTC process lengths fail to correlate with mitotic zone length [17], [19].

Here we analyze DTC architecture using myristoylated fluorescent proteins to label DTC membranes. We confirm known architectural features but discover that the extent of SIPs is greater than previously seen. We dub the striking collection of membranes in the distal mitotic zone the “DTC plexus”. This DTC plexus corresponds roughly to the undifferentiated GSC pool. We also find that maintenance of the plexus responds to the differentiation state of the germ cells. Possible functions of the plexus are discussed.

## Results and Discussion

### DTC architecture and discovery of the DTC plexus

To visualize DTC architecture, we used the *lag-2* promoter to drive expression of a fluorescent protein targeted to membranes with the Src kinase myristoylation tag (for example, myristoylated GFP [myr-GFP]). Focusing on young adult hermaphrodites (24 hours past mid-L4), we first confirmed DTC features seen previously (see Introduction). These features include the “cap” (Figure 1C–E), long external processes (LEPs) (Figure 1E), short intercalating processes (SIPs) (Figure 1D) and detached fragments (Figure 1D). We then measured the extent of each DTC feature along the distal-proximal axis using the standard metric of germ cell diameters (gcd) from the distal end (Figure 1B). Most measurements reported here confirmed previous observations [17], [19], but the SIPs were found to extend further from the DTC than seen previously (Figure 1D and 1F).

To check the validity of myr-GFP as a marker, we made a strain expressing myristoylated tdTomato (myr-tdTom) under the *lag-2* promoter and compared these distinct myristoylated fluorescent markers to each other and to cytoplasmic GFP (c-GFP), also under the *lag-2* promoter. We also compared the three markers (myr-GFP, myr-tdTom and c-GFP) in both intact, living animals and unfixed, dissected gonads. Together these experiments showed that DTCs have a similar architecture with all markers and under all conditions (Figure 1G, top two rows; data not shown).

We next quantitated myr-GFP fluorescent pixel intensity using projections of confocal z-series and the “Plot profile” function in ImageJ (Figure 1G, bottom row). The cap and LEPs were analyzed in a full projection of the approximately twenty 1 µz-slices that span the width of a single distal gonad. The cap coincided with the strongest peak of fluorescence in ImageJ (Figure 1E), and LEPs extended to where fluorescence drops to background (Figure 1E). Because SIPs localized internally, we scored them instead with a “core” projection of the central ten 1 µ slices of the distal gonad, and found that they extended to where fluorescence drops to background in this central region (Figure 1F). These results were confirmed with projections from 8 gonads (Figure 1G, compare all three rows).

We conclude that the DTC generates a distinct region, which we dub the DTC “plexus”. This plexus includes several DTC architectural features – the cap, SIPs, distal parts of the LEPs and distally localized internal fragments. The DTC plexus extends 8 to 9 gcd from the distal end. The extent of the plexus corresponds reasonably well with the GSC pool, which extends ∼6 to 8 gcd from the distal end [8].

Close and extensive contact of GSCs with somatic support cells is found in many systems [3]–[5]. For example, in fly ovaries, E-cadherin mediated adhesion is crucial for maintaining GSC contact with the niche [23]. In addition, fly ovarian escort cells have extensive contact with GSCs and it has been proposed that these escort cell processes help transfer germ cells to follicle cells [24]. In mammals, spermatogonial stem cells are encased in Sertoli cells though the precise role for this close association is not clear [3].

### The DTC plexus and GLD-1 expression

To examine the spatial relationship of the niche plexus with the state of germ cell differentiation in more detail, we examined the expression pattern of the differentiation regulator, GLD-1, relative to the niche plexus. The GSC pool has barely detectable GLD-1, but more proximal germ cells contain increasing GLD-1; the level of GLD-1 shifts from barely to easily detectable at a site corresponding to the end of the GSC pool [8], [14], [25].

**Figure 2:**
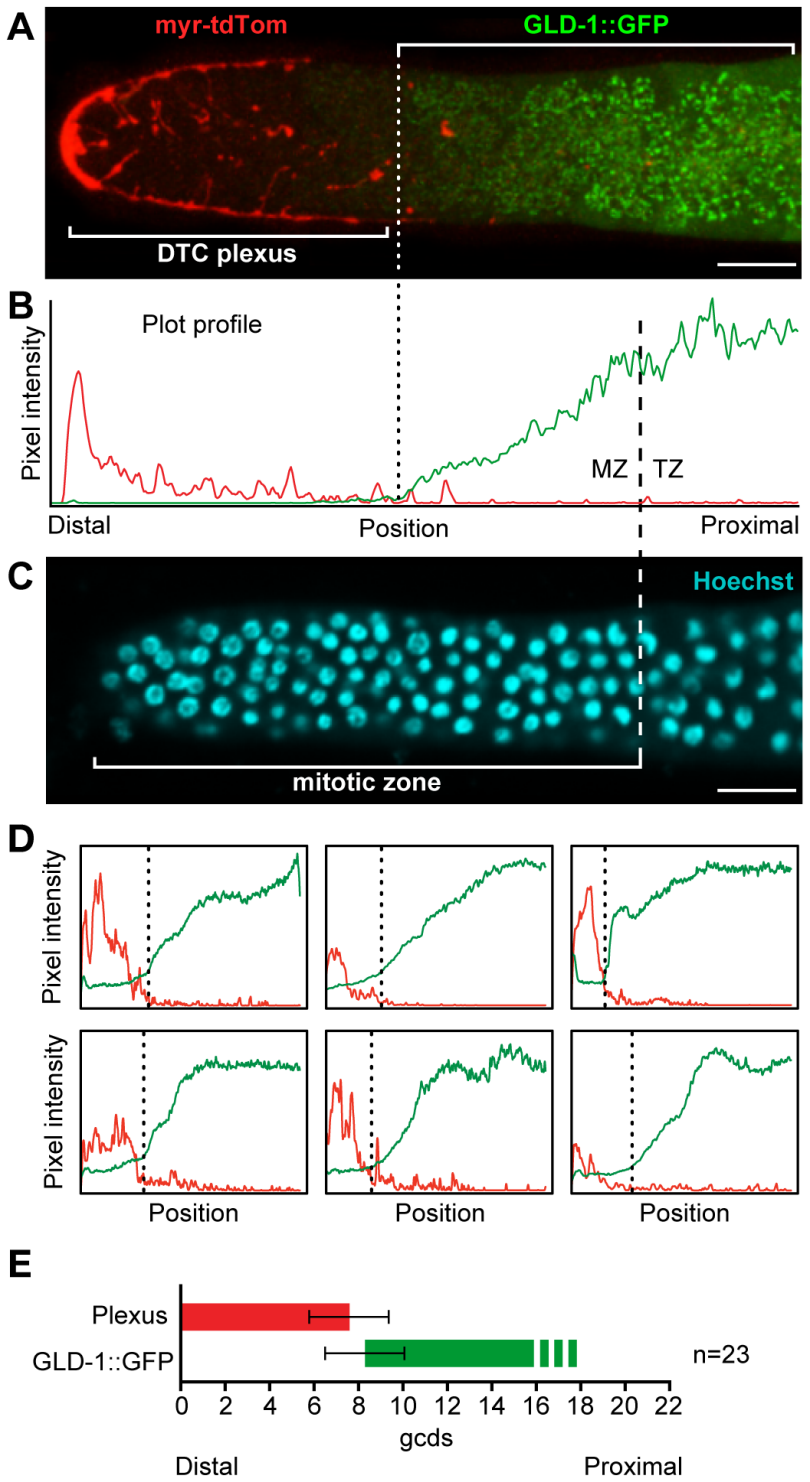
The DTC plexus ends where the differentiation marker, GLD-1, increases. (A) Core confocal projection of an extruded gonad showing the DTC plexus region (red) and GLD-1::GFP (green). The position where GLD-1 becomes easily detectable is marked by the dotted line. (B) Pixel intensity plots of myr-tdTom (red line) and GLD-1::GFP (green line). GLD-1::GFP intensity increases where myr-tdTom intensity drops to background, midway through the MZ. (C) Hoechst-stained germ cell chromatin (blue) shows proximal edge of MZ (dashed line). (D) Six additional germlines were scored using Plot profile as in (B). All 6 are similar to (B): myr-tdTom intensity drops to background where GLD-1::GFP intensity increases. (E) Average positions of DTC plexus and GLD-1::GFP (gcd±standard deviation). Scale bars in (A) and (C) = 10 µm.

We therefore asked if the plexus boundary corresponds to this shift in GLD-1 expression. To this end, we generated a strain expressing P*_lag-2_::myr-tdTom* and P*_gld-1_::GLD-1::GFP* (Figure 2A) and used ImageJ to quantitate both signals as described above (Figure 2B). When assessed individually using Plot profile, the plexus ended in the immediate region of the increase in GLD-1::GFP expression (n = 7; Figure 2B and 2D). When scored by eye, the plexus ended at or just distal to the increase in GLD-1::GFP in 83% of germlines (n = 23). When averaged, the plexus spanned 7.6±1.8 gcd from the distal end, while the increase in GLD-1::GFP expression occurred at 8.3±1.8 gcd from the distal end (Figure 2E). We conclude that the plexus corresponds to the region with barely detectable GLD-1 and therefore likely corresponds to the GSC pool. The correlation between the DTC plexus and the GSC pool is consistent with either a role for the plexus in maintaining the GSC pool or a role for the GSC pool in maintaining the plexus.

The membranes of the DTC plexus are likely to present GLP-1/Notch ligands to adjacent germ cells. The GLP-1/Notch ligands, LAG-2 and APX-1, are expressed in the DTC [16], [19], [22], [26] and are redundantly required for GSC maintenance (unpublished data) [16]. LAG-2 is found in the DTC processes as well as in the fragments [19], [22], at least some of which include internalized LAG-2 [26]. Whether this concentration of ligand amplifies GLP-1/Notch signaling in the plexus region remains to be determined.

The DTC plexus may also modulate GLP-1/Notch signaling. Multiple signaling pathways, including Notch, use filopodia as a mechanism to control the spatial distribution of membrane-bound signaling components. It has been proposed that these filopodia also allow quick responses to changing conditions [27]–[30], which may help cells respond to changes in environmental inputs. In addition, interaction between Notch ligands on dynamic filopodia of the signaling cells and the Notch receptor on receiving cells may provide physical tension that facilitates receptor cleavage and activation of Notch signaling [30].

### DTC architecture depends on germ cell state

We next asked if the germ cell state affects DTC architecture and the plexus in particular. The wild-type DTC plexus is found in the distal mitotic zone and hence in association with germ cells in the mitotic cell cycle. To ask if the plexus can be maintained when distal germ cells are manipulated to enter the meiotic cell cycle, we used *glp-1(q224ts)*, a temperature-sensitive allele of the GLP-1/Notch receptor. At permissive temperature (15°C), the *glp-1(q224ts)* mitotic zone is shorter than in wild type [8] but the distal germ cells remain in the mitotic cell cycle. By contrast, at restrictive temperature (25°C), all *glp-1(q224ts)* distal germ cells enter the meiotic cell cycle [31]. Moreover, when *glp-1(q224ts)* mutants are raised to adulthood at 15°C but shifted to 25°C as adults, their distal germ cells enter meiotic prophase within 6 hours [8].

**Figure 3:**
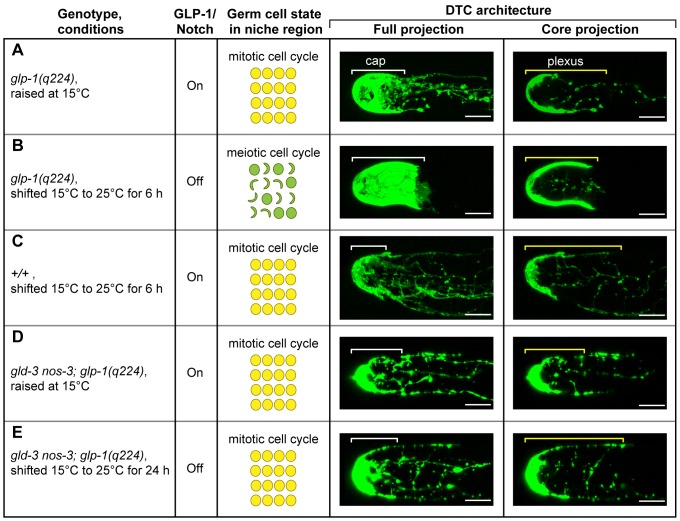
DTC architecture depends on germ cell state. (A–E) Extruded gonads from animals expressing myr-tdTom (A-C) or c-GFP (D,E). All fluorescence was pseudo-colored green for simplicity. Genotypes and growth conditions are shown in first column (see Methods for details); GLP-1/Notch activity is summarized in second column; germ cell state in the niche region is summarized in the third column (yellow, undifferentiated germ cells; green, germ cells in early stages of meiosis). Scale bar = 10 µm.

We examined DTC architecture in *glp-1(q224ts)* adults, either grown at permissive temperature or after shifting to restrictive temperature during adulthood. When grown at permissive temperature, the *glp-1(q224ts)* DTC architecture was similar to wild-type in most animals (91%, n = 22) (Figure 3A, similar to Figure 3C). In the remaining animals, DTC architecture was changed: the cap covered distal germ cells more extensively and SIPs were mainly found only under the cap (similar to Figure 3B). After the shift to restrictive temperature, the percentage of animals with altered DTC architecture went up dramatically with time, from 34% (n = 35) at 6 hours (Figure 3B) to 91% (n = 34) at 24 hours after the shift. As controls, we examined wild-type DTCs, which appeared normal at both 15°C (97%, n = 33) and 25°C (100%, n = 39) (Figure 3C; not shown).

The change in DTC architecture observed in *glp-1(q224ts)* adults shifted to restrictive temperature might have resulted from a loss of GLP-1/Notch signaling or from a loss of mitotic germ cells. To distinguish between these possibilities, we examined DTCs in the triple mutant, *gld-3 nos-3; glp-1(q224ts)*, which has a tumorous germline at both permissive and restrictive temperature. This tumorous germline results from a loss of the pro-differentiation regulators *gld-3* and *nos-3* [32], which function downstream of GLP-1/Notch so that germ cells are maintained in the mitotic cell cycle regardless of GLP-1/Notch signaling [33] (S. Millonigg and C.R. Eckmann, unpublished data). DTC architecture in *gld-3 nos-3; glp-1(q224ts)* adults was similar to wild-type when grown at permissive temperature (100%, n = 6; Figure 3D) and also when shifted to restrictive temperature (100%, n = 6; Figure 3E). Therefore, the change in DTC architecture seen in *glp-1(q224ts)* animals depends on germ cell state rather than on GLP-1/Notch signaling, suggesting the existence of feedback from germ cells to the niche. The dependence of support cell architecture on the differentiation state of the cells they interact with may be widespread. For example, escort cell architecture in the *Drosophila* ovary depends on the presence of differentiating germ cells [34]. This type of feedback to cellular architecture may help reinforce cell fate decisions and maintain the boundaries between self-renewing and differentiating cells.

### DTC plexus in mutants with altered mitotic zone lengths

We next asked whether the extent of the plexus correlated with the length of the mitotic zone. The DTC niche expresses at least two DSL ligands, LAG-2 and APX-1, which activate the GLP-1/Notch receptor in adjacent germ cells [16], [22]; Notch signaling maintains GSCs via FBF RNA-binding proteins, which are broad-spectrum inhibitors of differentiation; FBF represses expression of GLD proteins, which promote differentiation [12]. FBF refers to two nearly identical proteins, FBF-1 and FBF-2 [35], which are redundantly required for stem cell maintenance [36], but which are not equivalent [37]–[39]. Most relevant here, mutants in all these regulators can affect the length of the mitotic zone [36], [37]. However, effects of the mutants on the size of the GSC pool, contained within the mitotic zone (Figure 1A), are not known.

We analyzed the size of the DTC plexus in four mutants with altered mitotic zone size (Table 1). (1) The mitotic zone of *lag-2/+* heterozygotes was shorter than wild-type and plexus size was modestly but significantly shorter than wild-type. (2) The mitotic zone of *lag-2/+*; *apx-1*/+ double heterozygotes was yet shorter and the plexus was also shorter. (3) The mitotic zone of *fbf-1* mutants is shorter than wild-type [36], [37], but plexus size was not significantly different from wild-type. (4) The mitotic zone of *fbf-2* mutants is longer than wild-type [37] and, in this case as well, plexus size was not significantly different from wild-type.

**Table 1:** Extent of plexus in mutants with altered mitotic zone (MZ) lengths.

		DTC features (gcd)			
Marker	Genotype	Cap	Plexus	MZ	n
myr-GFP/+	*+/+*	4.0±1.1	8.6±1.8	19.1±2.3	36
	*lag-2/+*	4.0±0.8	7.6±1.8*	16.6±2.1***	32
	*lag-2 +/+ apx-1*	4.3±0.8	7.1±1.9***	13.0±2.9***	29
myr-GFP	*+/+*	3.8±0.8	8.3±2.2	17.7±2.1	31
	*fbf-1(ok91)*	3.8±0.9	7.4±2.5	15.3±2.6***	30
	*fbf-2(q738)*	3.7±1.1	7.4±1.8	19.8±2.8**	30

Asterisks show significance by unpaired t-test (*** P≤0.001, **P≤0.005, *P≤0.05).

These results suggest a correlation between DTC plexus length and mitotic zone length in animals with decreases in either one or two GLP-1/Notch ligands. At this point, it is not clear whether decreased ligand directly affects niche architecture or whether niche architecture responds to subtle changes in stem cell state or both. In *fbf* single mutants, we did not see a correlation between DTC plexus length and mitotic zone length, suggesting that, in these mutants, plexus size does not determine or respond to size of the mitotic zone. It is not clear why there is not a correlation between plexus size and mitotic zone length in *fbf* mutants. One possibility is that the GSC pool is the same size in *fbf-1* and *fbf-2* single mutants and that the differences in mitotic zone size reflect how robustly the stem cell regulatory network switches from a stem cell mode to a differentiating mode.

### DTC plexus forms at adulthood

**Figure 4:**
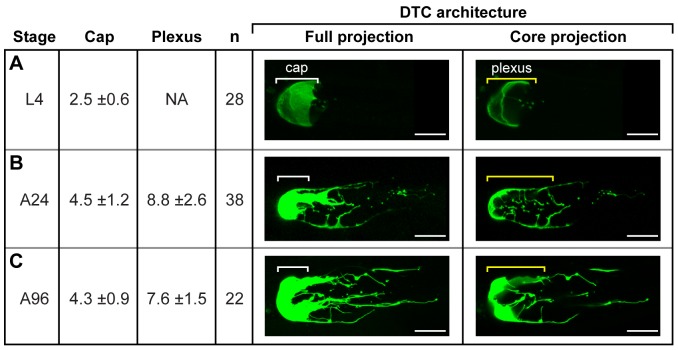
The DTC plexus forms in adults and is maintained through the reproductive period. (A–C) Extruded gonads from animals expressing myr-GFP. (A) L4. Pre-reproductive larva. These animals make only sperm; they do not produce embryos. The DTC has both leader and niche function at this stage and germ cell number is increasing. DTC architecture includes the cap and a few SIPs found associated with the cap. LEPs are not observed. (B) Reproductive adult, 24 hours after L4. The DTC functions as a stem cell niche [7]. Germ cell number is maintained. DTC architecture includes cap, extensive plexus and LEPs. (C) Post-reproductive adult, 96 hours after L4. Germ cell number is maintained and DTC architecture is similar to that seen in reproductive adults. Scale bar = 10 µm.

We examined DTC architecture from the fourth larval stage (L4) through reproductive adulthood (24, 48 and 72 hours past the mid-L4 stage) to ∼1-day into post-reproductive adulthood (96 hours past the mid-L4 stage; at this point most self-sperm have been depleted). Adults in this age range possess the same number of germline cells even though germ cells are continuously lost to cell death and gamete use [13], [19]. Using myr-GFP to examine DTC architecture, we found that L4 DTCs consist of a cap with few SIPs (Figure 4A); therefore, the plexus and long external processes have not formed. By early adulthood the cap has elongated and generated processes to form the plexus (Figure 4B); this plexus stays at approximately the same size from 24 to 96 hours (Figure 4C). Therefore, niche architecture changes dramatically between L4 and early adulthood but then stays essentially the same (also see [40]).

This developmental change in DTC architecture coincides with the time when the DTC loses its “leader” function: the larval DTC is not only a niche but also a migratory cell that leads gonadal elongation; by contrast, the adult DTC keeps its niche function but loses its migratory leader function [6], [41]. One attractive idea is that cellular machinery responsible for DTC migration [41]–[43] is rechanneled to drive DTC process formation at this stage.

## Conclusion

Using a myristoylated GFP to mark DTC membranes, we have observed extensive contact of this mesenchymal niche with adjacent germ cells. This contact occurs more extensively than previously observed and its extent correlates well with the GSC pool. The DTC plexus is newly discovered and we do not yet know its function. One possibility is that the plexus increases DTC-germ cell contacts to amplify or modulate the Notch signal across a cluster of germ cells. Another idea is that the plexus increases DTC-germ cell contacts to anchor germ cells at the distal end and prevent their proximal migration. And a third suggestion is that it creates a microenvironment that has not yet been discovered (e.g. change in oxygen tension or nutrient availability). These ideas are of course not mutually exclusive and remain highly speculative. Regardless, the correspondence of the DTC plexus and GSC pool is suggestive and likely to play a role in GSC regulation.

## Materials and Methods

### Strains and genetics

All strains were maintained at 20°C as described [44] unless otherwise noted. We used the wild-type Bristol strain N2 as well as the following mutants: LGII: *fbf-1(ok91)* [36], *fbf-2(q738)* [37], *nos-3(q650)* [45], *gld-3(q730)* [46]; LGIII: *glp-1(q224)* [31]; LGV: *lag-2(q411)* [22], *him-5(e1490)* [47], *apx-1(or3)* [16]. Transgenes included the P*_lag-2_::c-GFP* (*qIs19*, *qIs56*, and *qIs57*) transcriptional reporter [48], P*_lag-2_::myr-GFP* (*qIs153*) (this paper), P*_lag-2_::myr-tdTomato* (*qIs154*) (this paper), and the rescuing translational reporter P*_gld-1_::GLD-1::GFP* (*ozIs5*) (same construct as *ozIs2* [49]).

### Construction of fluorescent protein markers

myr denotes the addition of a myristoylation sequence from Src kinase (SRC-1), which associates with membranes [50]; in these transgenes, myr is fused to the N-terminus of either GFP or tdTomato, a red fluorescent protein [51].

P*_lag-2_::myr-GFP* was made by adding the 5′ sequence of *src-1* (Y92H12A.1) encoding its myristoylation sequence to a 5′ primer sequence used to amplify GFP and the *unc-54* 3′ UTR from pPD95.81 (kindly provided by A. Fire):

DB37 = AggtaccaataaataATGGGTTGCCTGTTTTCAAAAGAGCGGCGAAGTAAAGGAGAAGAACTTTTC

DB10 = aaccgcgggcggccgcaagcttGATAAGGTATTTTGTGTGCGG

P*_lag-2_::myr-tdTomato* was made by adding the 5′ sequence of *src-1* (Y92H12A.1) encoding its myristoylation sequence to a 5′ primer sequence used to amplify tdTomato and the *unc-54* 3′ UTR from a tdTomato version of pPD95.81 (gift of Barr lab):

DB122 = AggtaccaataaataATGGGTTGCCTGTTTTCAAAAGAGCGGCGAgtgagcaagggcgaggaggtc

DB22 = agcggccgcCGGCCGACTAGTAGGAAACAG

All fluorescent protein markers were generated by injecting the plasmid DNA of interest at 5 ng/µL into N2 animals using either P*_ttx-3_::RFP* or P*_ttx-3_::GFP* at 5 ng/µL as a coinjection marker. Transgenes were integrated by UV/trimethylpsoralen mutagenesis and backcrossed against N2 at least four times.

### Scoring distal tip cell features

#### Live animals by compound fluorescence microscopy and Nomarski optics

Live animals were immobilized with 0.25 mM levamisole in M9 on a glass slide with a 2% agarose pad and observed using a 63× Plan-apochromat objective and appropriate filter set (Chroma, Battleboro, VT) on a Zeiss Axio Imager D1 microscope. The DTC cap was scored by noting the most proximal extent of myr-XFP covering the surface of germ cells, the DTC plexus was scored by noting the most proximal process of the DTC intercalating between germ cells perpendicularly from the gonad surface, and DTC long external processes (LEPs) were scored by noting the most proximal intact membranous process along the exterior of the gonad. Lengths of the cap, plexus, and LEPs were scored by switching to Nomarski optics and counting the number of germ cell diameters along the distal-proximal axis to each point described above. Images were obtained with a Hamamatsu Orca digital camera (Bridgewater, NJ) using Improvision Openlabs software (Lexington, MA). Images were processed in Adobe Photoshop (San Jose, CA).

#### Unfixed extruded gonads by compound fluorescence microscopy and Nomarski optics

Gonads were dissected in 0.25 mM levamisole in M9. In some cases, Hoechst 33342 (Invitrogen) was added to the dissection media at 100 ng/mL. Dissected gonads were observed as described above. For gonads stained with Hoechst 33342, lengths of the DTC cap, plexus, and LEPs were scored by counting germ cell nuclei visualized by the Hoechst fluorescence, and the proximal boundary of the mitotic zone was scored as described below.

#### Unfixed extruded gonads by confocal laser scanning microscopy

Gonads were dissected in M9 with 0.25 mM levamisole and 100 ng/mL Hoechst 33342 (Invitrogen). Images were collected using a 63× objective on a Zeiss LSM510 Meta laser scanning confocal microscope. Full projections of z-stacks containing approximately twenty 1 µm sections were made using ImageJ [1.41o] [52]. Core projections were made using the central ten 1 µm sections that did not include the top and bottom surfaces of the cap. The lengths of the cap and LEPs were measured using the full maximum intensity projection and counting the number of germ cell nuclei along the distal-proximal axis to the proximal edge of the cap and longest LEP. The plexus was measured using the core projection and counting the number of germ cell nuclei to the point where fluorescence drops to background levels.

#### Plot profile analysis in ImageJ

Full and core (middle 10 µm) projection images were analyzed in ImageJ using “Plot profile”. For Plot profile, a box was drawn around the gonad so that the left side aligned with the distal edge of the DTC; pixel intensity was calculated within this area. The proximal edge of the cap is defined by the first drop in the pixel intensity profile along the distal-proximal axis of a full projection image. The longest LEP is defined by the point along the distal-proximal axis at which the pixel intensity drops to background in a full projection image. The plexus is defined by the point along the distal-proximal axis at which the pixel intensity drops to background in the core projection image. To convert the lengths of the cap, plexus, and LEPs defined by their pixel profile to germ cell diameters, we determined the length between the distal edges of 2 germ cell nuclei in pixels (∼20 pixels per germ cell diameter) and divided the length of the DTC features in pixels by the number of pixels per germ cell diameter.

### Determination of the mitotic zone/transition zone boundary

The mitotic zone/transition zone boundary was scored as described previously [19]. Briefly, the mitotic zone/transition zone boundary was defined as the distal-most row of cells containing multiple nuclei with crescent-shaped DAPI or Hoechst morphology, which is typical of leptotene/zygotene of meiotic prophase I [53], [54].

### Alteration of germ cell state to determine DTC morphology

To measure DTC morphology in response to germ cell state change, the following strains were maintained at 15°C (permissive temperature for *glp-1(q224)*): JK4478: *ozIs5 I; glp-1(q224) III; him-5(e1490) qIs154 V*; JK4406: *ozIs5 I; him-5(e1490) qIs154 V*; JK4553: *gld-3(q730) nos-3(q650)/mIn1 II; glp-1(q224) III; qIs56 V*; JK4552: *gld-3(q730) nos-3(q650)/mIn1 II; qIs56 V*. Animals were picked at mid to late L4 to new plates and placed back at 15°C for 24–28 hours (to young adult stage). Plates were then either left at 15°C or shifted to 25°C (restrictive temperature for *q224*) for another 6, 9, 12 or 24 hours. DTCs were scored in unfixed extruded gonads stained with Hoechst 33342 by either compound fluorescence or confocal microscopy.

### Staging animals for examining DTC in mitotic zone size mutants

To examine DTC morphology in mutants affecting mitotic zone size, mid to late L4 animals were picked to new plates and placed back at 20°C for 24 hours (to young adult stage). DTCs were scored in unfixed extruded gonads stained with Hoechst 33342 by either compound fluorescence or confocal microscopy.

### Staging animals for examining DTC during development

To examine DTC during development, mid to late L4 animals were either dissected in M9 with 0.25 mM levamisole and 100 ng/mL Hoechst 33342 to image L4 DTCs or picked to new plates and placed back at 20°C. Animals were then picked for dissection at 24 or 96 hours after mid to late L4. DTCs were scored in unfixed extruded gonads stained with Hoechst 33342 by either compound fluorescence or confocal microscopy.
